# A novel *Plasmodium vivax* vaccine based on recombinant chimpanzee adenovirus ChAd63 and MVA expressing TRAP

**DOI:** 10.1186/1475-2875-11-S1-O49

**Published:** 2012-10-15

**Authors:** Karolis Bauza, Oliver Billker, Adrian VS Hill, Arturo Reyes-Sandoval

**Affiliations:** 1 The Jenner Institute, University of Oxford, Oxford, Oxfordshire, OX3 7DQ, UK; 2 Wellcome Trust Sanger Institute, Hinxton, Cambridge, Cambridgeshire, CB10 1SA, UK

## Background

*P. vivax* is the most geographically widespread human malaria and is considered to be the most prevalent form in some regions of Latin America, Central and South-East Asia, accounting for up to 390 million clinical infections every year and an estimated 2.6 billion people being at risk of infection with *P. vivax *[1,2]. An effective vaccine against this protozoan would have a major global impact on the disease burden [3]. Modified Vaccinia Ankara (MVA) and the chimpanzee adenovirus ChAd63 are two clinically relevant viral vectors that have been shown to induce strong and protective antibody and T-cell responses against *P. falciparum* TRAP, both in pre-clinical studies and clinical trials [4-7].

## Materials and methods

We developed recombinant ChAd63 and MVA vectors expressing *P. vivax* TRAP (PvTRAP), which were used to assess T-cell and antibody responses upon sequential immunisation (prime-boost) of mice. Vaccine efficacy was assessed through challenge with a newly developed transgenic *P. berghei* expressing PvTRAP.

## Results

High antibody titres and frequencies of PvTRAP-specific T cells were induced in all tested inbred and outbred mouse strains. The newly developed parasite showed similar fitness to wild type *P. berghei* and was successfully used to infect and assess protection in vaccinated mice. The Ad-MVA prime-boost regimen induced good protective levels regardless of the mouse strain.

## Conclusions

The strong immunogenicity and protective efficacy elicited by the recombinant ChAd63 and MVA viruses expressing PvTRAP indicate that this vaccine approach has a good potential to be tested in clinical trials in the near future.
